# An Unusual ECG Artifact Caused by Faulty Cardiac Monitor Leads

**DOI:** 10.5811/cpcem.2021.3.51694

**Published:** 2021-04-23

**Authors:** Bryan Imhoff, Wesley Casey

**Affiliations:** University of Kansas Health System, Department of Emergency Medicine, Kansas City, Kansas

**Keywords:** ECG, artifact, emergency department, cardiac monitor

## Abstract

**Case Presentation:**

We present the case of a 74-year-old female patient who presented to the emergency department with lower extremity weakness found to have a fixed frequency square wave artifact in all leads of her electrocardiogram (ECG). After troubleshooting, faulty external cardiac monitor leads were identified as the cause of this unique artifact.

**Discussion:**

The ECG is an important diagnostic tool for medical providers. Electrocardiogram artifacts are extremely common, and knowledge of artifacts is necessary to prevent inappropriate interpretation, diagnostic error, and unnecessary workup. Medical providers should have a low threshold for suspicion when ECG findings do not correlate with the patient’s chief complaint or history of present illness. They must also be familiar with the most frequent ECG artifact variants and be prepared to follow a stepwise approach to troubleshoot less frequent variants.

## CASE PRESENTATION

**Image 1:**
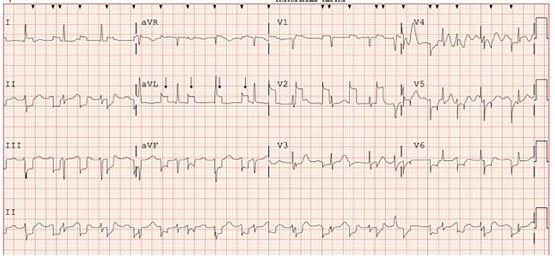
Electrocardiogram, demonstrating a fixed frequency square wave artifact (arrows).

**Image 2:**
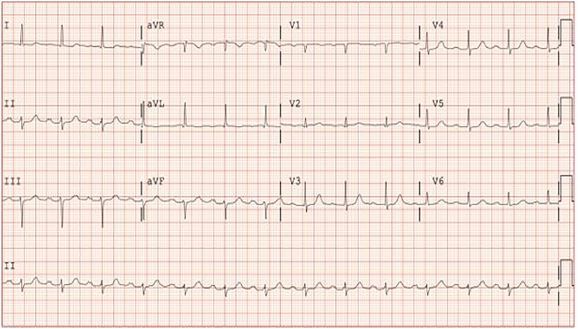
Repeat electrocardiogram, demonstrating resolution of artifact after exchanging cardiac monitor leads.

A 74-year-old female presented to the emergency department for progressive left leg weakness that had resulted in multiple falls. An electrocardiogram (ECG) was obtained per Image 1. The ECG had a fixed frequency square wave artifact in all leads, not present on previous ECGs. The patient reported no implanted pacemaker and no neural stimulator, but he was wearing a BioTel event monitor (BioTelemetry, Inc, Malvern, PA), which had been placed in the prior month for syncope. The nurse turned off and removed the event monitor and replaced the ECG machine main cable. A repeat ECG was unchanged. The nurse then exchanged the room’s cardiac monitor leads. A repeat ECG showed complete resolution of the artifact, shown in Image 2. The initial set of cardiac monitor leads showed no visible damage but appeared to be the cause of this unique artifact.

## DISCUSSION

Electrocardiogram artifact is common and can be generated by physiologic and non-physiologic causes as summarized in the table below.^1^,^2^ Studies of the effect of electromagnetic interference (EMI) on medical devices represent a large body of literature. For example, due to their ubiquity, cell phones have been extensively studied and identified as a source of potential EMI, including their effect on ECG capture.^3^ The effects of EMI on implanted devices, particularly cardiac pacemakers, have also been studied extensively due to the potential of negative outcomes from device malfunction.^4^,^5^ Malfunction of an ECG machine cable is documented in the literature as a potential cause of ECG artifact; however, to the best of our knowledge no case studies exist demonstrating a separate external cardiac monitor, or its leads, causing ECG interference and artifact. Medical practitioners must have a low threshold for suspicion when ECG findings do not correlate with the patient’s chief complaint or history of present illness. In the presence of a presumed artifact, the practitioner should employ a stepwise approach to isolating the source.

**Educational Merit Capsule**

What do we already know about this clinical entity?

*Electrocardiogram (ECG) artifact as a general topic has been studied extensively.*

What is the major impact of the image(s)?

*This image represents a new, not previously documented, case of ECG artifact, and the accompanying discussion presents a more holistic list of other potential causes of artifact.*

How might this improve emergency medicine practice?

*Recognition of ECG artifact is necessary to prevent inappropriate interpretation, diagnostic error, and unnecessary workup in the emergency department.*

## Figures and Tables

**Table:** Categories and sources of electrocardiogram artifact.

Category	Source of artifact
Physiologic	Patient muscle activity Patient motion
Non-physiologic	Electromagnetic interference Implanted medical devices (eg. electrostimulators) Nearby electrical medical devices (eg, hemodialysis machines, cardiopulmonary bypass machines, ventilators, intravenous fluid warmers, endoscopes, temperature monitors, irrigation pumps, electrocauteries, magnetic resonance imaging machines) Other non-medical devices (eg, light fixtures, cell phones) Cable and electrode malfunction Insufficient amount of electrode gel Fractured wires Inappropriate filter settings Loose connections Misplaced leads Accumulation of static energy
